# One session of fMRI-Neurofeedback training on motor imagery modulates whole-brain effective connectivity and dynamical complexity

**DOI:** 10.1093/texcom/tgac027

**Published:** 2022-07-25

**Authors:** Eleonora De Filippi, Theo Marins, Anira Escrichs, Matthieu Gilson, Jorge Moll, Fernanda Tovar-Moll, Gustavo Deco

**Affiliations:** Computational Neuroscience Group, Center for Brain and Cognition, Department of Information and Communication Technologies, Universitat Pompeu Fabra, Barcelona, Carrer de Ramon Trias Fargas, 25-27, 08005 Barcelona, Catalonia, Spain; D’Or Institute for Research and Education (IDOR), Rua Diniz Cordeiro 30, Botafogo-Rio de Janeiro, 22281-100, Brazil; Post-Graduate Program in Morphological Sciences, Institute of Biomedical Sciences, Federal University of Rio de Janeiro, Citade universitaria da Universidade Federal do Rio de Janeiro, 21941-590, Brazil; Computational Neuroscience Group, Center for Brain and Cognition, Department of Information and Communication Technologies, Universitat Pompeu Fabra, Barcelona, Carrer de Ramon Trias Fargas, 25-27, 08005 Barcelona, Catalonia, Spain; Computational Neuroscience Group, Center for Brain and Cognition, Department of Information and Communication Technologies, Universitat Pompeu Fabra, Barcelona, Carrer de Ramon Trias Fargas, 25-27, 08005 Barcelona, Catalonia, Spain; D’Or Institute for Research and Education (IDOR), Rua Diniz Cordeiro 30, Botafogo-Rio de Janeiro, 22281-100, Brazil; D’Or Institute for Research and Education (IDOR), Rua Diniz Cordeiro 30, Botafogo-Rio de Janeiro, 22281-100, Brazil; Post-Graduate Program in Morphological Sciences, Institute of Biomedical Sciences, Federal University of Rio de Janeiro, Citade universitaria da Universidade Federal do Rio de Janeiro, 21941-590, Brazil; Institució Catalana de la Recerca i Estudis Avançats (ICREA), Passeig de Lluis Companys, 23, 08010, Barcelona, Catalonia, Spain; Department of Neuropsychology, Max Planck Institute for human Cognitive and Brain Sciences, Stephanstrasse 1a, 04103, Leipzig, Germany; Turner Institute for Brain and Mental Health, Monash University level 5, 18 Innovation Walk, Clayton Campus. Wellington Road, Clayton VIC 3800, Australia

**Keywords:** neurofeedback, real-time fMRI, motor imagery, whole-brain effective connectivity, whole-brain dynamics

## Abstract

In the past decade, several studies have shown that Neurofeedback (NFB) by functional magnetic resonance imaging can alter the functional coupling of targeted and non-targeted areas. However, the causal mechanisms underlying these changes remain uncertain. Here, we applied a whole-brain dynamical model to estimate Effective Connectivity (EC) profiles of resting-state data acquired before and immediately after a single-session NFB training for 17 participants who underwent motor imagery NFB training and 16 healthy controls who received sham feedback. Within-group and between-group classification analyses revealed that only for the NFB group it was possible to accurately discriminate between the 2 resting-state sessions. NFB training-related signatures were reflected in a support network of direct connections between areas involved in reward processing and implicit learning, together with regions belonging to the somatomotor, control, attention, and default mode networks, identified through a recursive-feature elimination procedure. By applying a data-driven approach to explore NFB-induced changes in spatiotemporal dynamics, we demonstrated that these regions also showed decreased switching between different brain states (i.e. metastability) only following real NFB training. Overall, our findings contribute to the understanding of NFB impact on the whole brain’s structure and function by shedding light on the direct connections between brain areas affected by NFB training.

## Introduction

“The great, growling engine of change: technology” wrote the American writer Alvin Toffler in his book “Future shock” almost 5 decades ago (1970). While already at the end of the XX century technology was used to train humans to self-regulate their own brain activity (Kamiya 1968; Sterman and Friar 1972; Sterman and Wyrwicka 1967), now than ever before technology is the driving force of change, including the change of our own brain. In the past decades, noninvasive brain stimulation techniques have been widely used to promote beneficial brain changes under healthy and pathological conditions, and to investigate the relationship between brain activity and behavior (Begemann et al. 2020; Brunoni and Vanderhasselt 2014; Cotelli et al. 2020; Kan et al. 2020; Liu et al. 2019). In this way, neurofeedback (NFB) by functional magnetic resonance imaging (fMRI) has emerged as a promising tool to drive brain plasticity via closed-loop brain training (Sitaram et al. 2017). Previous studies have shown that with appropriate training, healthy individuals can learn to modulate the activity of brain areas involved in motor control (Berman et al. 2012; He et al. 2020; Marins et al. 2019; Scharnowski et al. 2015), language processing (Rota et al. 2009; Sreedharan et al. 2019; Zweerings et al. 2019), and emotional regulation (Lorenzetti et al. 2018; Moll et al. 2014; Zotev et al. 2013 for a review see Linhartová et al. 2019), together with the balance of activity between brain hemispheres (Chiew et al. 2012; Neyedli et al. 2016), and functional and effective connectivity (EC) of distant brain regions (Koush et al. 2013, 2017).

In order to provide a better characterization of the neural processes underlying NFB training, several studies have explored its effects on resting-state networks. For example, it has been shown that a single session of NFB-training targeting sensorimotor brain areas led to strengthening of both sensorimotor and default-mode networks (DMNs) (Marins et al. 2019). Increased functional connectivity (FC) of the empathy networks has also been described after modulation of anterior insula (Yao et al. 2016). In patients with major depressive disorder, amygdala-targeted NFB training led to amygdala FC changes during resting state (Young et al. 2018). However, these measurements do not provide inference about the coupling between brain areas, yielding limited information about impact of NFB training on brain networks. In fact, FC analysis only captures the level of correlated activity between pairs of brain areas, thus not allowing to describe the causal influence of information transmission from one given area to another, nor the simultaneous influence via third-party areas.

On the other hand, the use of whole-brain computational modeling to explain neuroimaging data has the potential of providing mechanistic information underlying different brain states in health and diseases, and as well as treatments monitoring. A recent modeling approach, the “MOU-EC” (Gilson et al. 2016, 2020), allows to estimate EC by linking structural connectivity (SC) with whole-brain functional dynamics. In particular, this approach uses the underlying SC to force the dynamical model to reproduce FC dynamics by modulating structural connections and explain them by the structure of the EC. In this sense, EC gives a measurement of the direct influence that one brain area may exert on others (i.e. how information propagates), thus providing information about the causal processes underlying brain functional dynamics. EC profiles estimated through the MOU-EC model have been shown to provide reliable biomarkers for subjects identification and for different cognitive tasks (Pallares et al. 2018; Senden et al. 2018), and a mechanistic understanding of mental illness (Rolls et al. 2018), brain disease (Adhikari et al. 2020), and developmental disorders (Rolls et al. 2020).

In this study, we interrogate data of a previous randomized, double-blind, and sham-controlled study (Marins et al. 2019) in order to explore the functional relevance of training-related structural plasticity through EC, going beyond conventional FC analysis. Specifically, we sought to (i) identify changes in information propagation (i.e. EC) that can predict the type of NFB training (real vs. sham) delivered, (ii) extract training-related signatures by Recursive Feature Elimination the support-network (ROI to ROI links) that contributed to the successful classification, and (iii) investigate through a data-driven approach how differences in information propagation are reflected in the functional dynamics following training, both at the whole-brain level and within the support network. We hypothesized that EC measures would have been a better predictor as compared with standard FC to discriminate both resting-state sessions and groups (i.e. NFB and control group), as they hold information about the underlying anatomical connectivity. Furthermore, we expected to find changes in brain’s spatiotemporal dynamics for the NFB group but not for the control subjects who underwent sham training. To the best of our knowledge this is the first study investigating whole-brain causal effects of NFB by means of estimated EC and machine-learning techniques.

## Materials and methods

### Participants

As described before in Marins et al. (2019), 38 healthy adults participated in the present double-blinded study, and were randomly assigned to compose either the real (NFB) or sham (CTL) group. Sample size has been estimated based on pilot data. Five participants displayed excessive ($\geq $2 mm) head movements for more than 25 percent of the acquisition and were excluded from our analysis. As a result, 16 participants composed the CTL group (7 males, mean age: 27.3 years, SD: 5.8) and 17 participants the NFB group (6 males, mean age: 27.4 years, SD: 3.5). The study has been approved by the D’Or Institute Ethics and Scientific Committee and conducted in accordance with the ethical standards compliant with the Declaration of Helsinki.

### Experimental Design

**Fig. 1:**
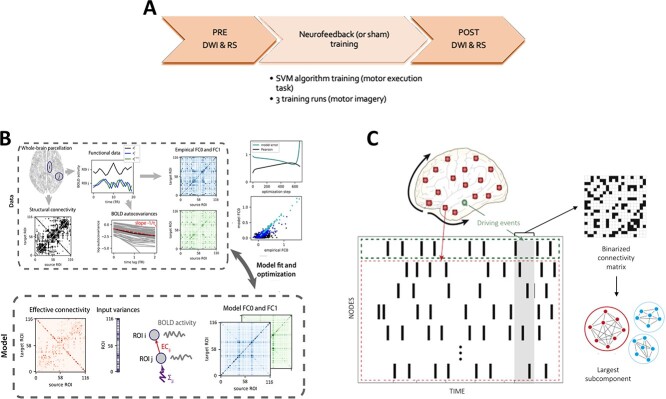
**Methods.** **A) Experimental design:** Immediately before (PRE) and after (POST) NFB training, resting-state and diffusion-weighted imaging (DWI) data were acquired. The training started with a motor execution task in which a 2-class support vector machine (SVM) algorithm was trained to discriminate between motor execution and rest based on the distributed voxel patterns. The NFB (or sham) training included 3 runs, in which participants performed a motor imagery task with the aid of the NFB (either real or sham) to recruit brain areas associated with motor execution obtained previously. **B) MOU-EC model workflow (adapted from Gilson et al. 2019):** The estimation of model parameters is done using the resting-state functional data parcellated into 116 ROIs and the SC connectivity matrix (black and white matrix on the left), with the latter used as a binary matrix to constrain the model’s topology. From the resting-state timeseries the empirical BOLD autocovariance matrices are calculated (blue and green matrices on the right of top panel) both with and without time lag (FC1 and FC0, respectively), and then reproduced by the model. The model’s estimation of the autocovariance matrices and effective connectivity matrix undergoes the Lyapunov optimization procedure using a gradient descent algorithm which minimizes the model error between the model’s estimate and the empirical FC0 and FC1.The iterative optimization procedure is repeated until the best fit between the model’s estimate and the empirical FC matrices is reached (high Pearson correlation coefficient), thus reducing the model error to the minimum (green and black lines in the left upper panel of Fig. 1B. **C) Algorithm to calculate the intrinsic ignition. Adapted from**Deco and Kringelbach (2017): The process to calculate the intrisic ignition involve the following steps: -Spiking neurons (green dot) produce driving events, which are captured applying a threshold as explained in Tagliazucchi et al. (2012). - For each driving event (gray area), the activity in the rest of the network is calculated within the time-window of 4TR (the whole red area). -A binarized matrix (black-and-white matrix on the right) is constructed for each time window according to the reoccurrence of driving events from different brain areas. -The largest subcomponent (blue and red graphs at the bottom) of the binary matrix represents a measure of global integration. -The previous steps are repeated for each driving event, thus allowing to calculate the mean and the variability of the Intrinsic-Driven integration of each brain area.

In brief, the study consisted in a single-day NFB (or sham) training, which started (PRE) and ended (POST) with consecutive acquisitions of diffusion-weighted imaging (DWI) and resting-state (RS) (Fig. 1A).

Before the training started, participants were asked to perform an overt motor task of a predefined sequence of right-hand finger movements (4-2-3-1-3-4-2, in which 4 is the little finger, 2 is the middle one, 3 is the ring, and 1 is the index), which served to train a 2-class support vector machine (SVM) algorithm to discriminate between motor execution and rest based on a distributed voxel patterns during the following NFB training. Brain areas involved in motor learning (Hardwick et al. 2013) were used as feature selection to restrict SVM. Next, in the 3 NFB (or sham) training runs participants performed a motor imagery task of the same predefined movement while watching a dynamic bar graph that varied according to the accuracy in which the SVM algorithm detected motor executed-related brain pattern (instead of rest brain pattern). Sham training was delivered to the CTL group in a double-blinded way based on the neurophysiological signals from a previous participant of the NFB group. Both motor execution and imagery tasks consisted of a block design of “GO” ( 20 s-long) and “STOP” (relax, 20 s-long), repeated 8 times. The complete description of the experimental design can be found in the previous publication (Marins et al. 2019). All the real-time data analysis and stimuli delivery were performed using F.R.I.E.N.D. toolbox (Sato et al. 2013). We used an acceleration sensor attached to the distal phalanx of the right middle finger throughout the whole experiment, which showed that motor imagery has been performed with no overt hand movements, similarly in both groups.

### MRI Data Acquisition

The study was performed with a 3T Achieva scanner (Philips Medical Systems, the Netherlands) using an 8-channel SENSE head coil. DWI (2.5mm x 2.5mm x 2.5mm, no gap, TR/TE (ms) = 5582/65, FOV = 240 x 240, matrix = 96 x 95) with diffusion sensitization gradients applied in 64 noncollinear directions, with a b factor of 1000 s/$mm^{2}$. Functional images were obtained with a single-shot T2-weighted echoplanar imaging (EPI) sequence (TR = 2000 ms, TE = 22 ms, matrix 80x80, FOV isotropic, no gap, 40 slices) 200 (task-based, either ME or MI) or 240 (resting-state), 240 x 240 x 120 mm, flip angle= 90${\circ }$, voxel size 3mm (resting state) volumes long. Before each functional imaging, 5 dummy volumes were collected for T1 equilibration purposes. Reference anatomical images were acquired using a T1-weighted 3-dimensional magnetization-prepared, rapidly acquired gradient echo (MP-RAGE) 1mm isotropic voxel size, 170 sagital slices). Head motion was restricted with foam padding and straps over the forehead and under the chin. The total MRI acquisition lasted about 60 min.

### Resting-state fMRI preprocessing

We computed preprocessing of functional resting-state data using the Data Processing Assistant for Resting-State fMRI (DPARSF; Yan and Zang 2010) based on SPM (Wellcome Trust Center for Neuroimaging, London, England).

First, we manually reoriented both functional and anatomical images. Then, resting-state images were preprocessed as follows: removal of the first 5 time-points, motion correction through realignment across volumes, co-registration of fMRI images to T1-weighted images by applying unified segmentation, correction for head movements parameters, for global mean signal, for the white-matter, and for cerebrospinal fluid signal by means of a regression of nuisance covariates. Further steps involved spatial normalization in stardard MNI (Montreal Neurological Institute) space, to 3$\times $3$\times $3 mm isovoxels, smoothing using a 6-mm full-width-at-half-maximum Gaussian kernel and a band-pass temporal filtering of 0.01–0.25 Hz. Finally, BOLD time-series were extracted for 100 cortical regions using the 7-Networks Schaefer Parcellation (Schaefer et al. 2018) and 16 subcortical regions using the Melbourne subcortical functional parcellation (Tian et al. 2020).

### Probabilistic Tractography analysis

Whole brain SC was performed individually in the native space using FMRIB’s Diffusion Toolbox (FDT; Behrens et al. 2003). First, non-brain tissue was removed, eddy current and head motion were corrected. Then the probabilistic model was fitted on corrected data using BEDPOSTX. Probability tractography was performed in a standard way using PROBTRACKX. Regions of interest were defined based on the 7-Networks Schaefer Parcellation (Schaefer et al. 2018) and 16 subcortical regions from the Melbourne subcortical functional parcellation (Tian et al. 2020). ROIs in the MNI space were co-registered to the native space using a nearest neighbourhood approach. SC between every pair of ROIs was calculated as the number of streamlines seeded in one ROI that reached another ROI. The mean connectivity between each pair of ROIs was calculated as the average of both bidirectional values.

### Whole-brain EC model: MOU-EC

We applied a dynamic generative model, the MOU-EC, to extract whole-brain connectivity estimates from the BOLD time-series of both resting-state sessions of NFB and CTL groups.

#### Parameters estimation

The whole parameter estimation procedure is presented in Fig. 1B. First, we constrained the model’s topology by applying a threshold to the average probabilistic SC matrices in order to maintain only a 30% density of structural pathways. The choice to preserve a density of anatomical pathways around 30% was made to match the density of SC at similar parcellations and it was based on the fact that we wanted to focus only on strong weights of structural pathways. In this way, we kept the weights for nonexistent connections (i.e. lower values than the fixed threshold) to 0, while the weights of above-threshold structural links were estimated from the functional matrices. We used the average probabilistic SC matrix of all participants for each group when comparing fMRI resting-state sessions before and immediately after the training within each group. Since a previous study published with this dataset (Marins et al. 2019) found structural changes in the NFB group but not in the control group, we used the intersected SC matrix (i.e. retaining only the common connections that were above the threshold in NFB and CLT subjects) when comparing both groups. Then, we applied a bandpass filter with the narrowband 0.01–0.0.07 Hz to the resting-state fMRI data, and we calculated the BOLD autocovariance both with (FC1) and without time lag (FC0). The autocovariance matrices was then reproduced by the model, which is based on a dynamic system with linear feedback, the Multivariate Ornstein–Uhlenbeck (MOU) process. The mathematical formula that describes the MOU process is

$$\begin{align*} dx_{\textrm{i}} = \left(\frac{-x_{i}}{T_{x}}+ \sum_{j\neq i}C_{\mathrm{ji}} x_{j}\right)dt+dB_{i} \end{align*}$$

in which $x_{\textrm{i}}$ indicates the activity of the brain region *i,* which is affected by the activity of other regions, and show an exponential decay by the time constant $T_{x}$. The matrix $C_{\textrm{ji}}$ stores information about the EC between 2 brain regions (*i,j*), and its topology is constrained by the SC mask after applying a threshold to retain only the strongest connections, as explained above. Here, $dB_{i}$ refers to the diagonal of the covariance matrix $\Sigma $, which holds information about the node local variability. Therefore, the model estimates of FC are given by the propagation of the local variability that generates network feedback through EC. In the MOU process, both the EC matrix $C_{\textrm{ji}}$ and the model’s parameter $\Sigma $ undergo a Lyapunov optimization procedure to minimize the model’s error at each optimization step. The aim of this iterative gradient-descent optimization is to tune the model to reproduce the empirical BOLD covariance matrices, both zero-lagged (FC0) and time-lagged (FC1), and the estimated EC with the minimum error and the maximum values of Pearson correlation coefficient mean for each session. The complete mathematical details about the model are described in Gilson et al.(2016, 2020). The code of the parameter estimation procedure is available at github.com/MatthieuGilson/WBLEC_toolbox.

#### Machine-learning approach

We performed both within- and between-group classification using the scikit-learn package (Pedregosa et al. 2011) based on Python language. First, we classified pre- and post-training (real or sham) resting-state sessions within each group. Then, we compared post-training resting-state session between subjects who underwent NFB training and subjects who received the sham training (i.e. CTL group). We constructed the feature arrays of both NFB and CTL groups using the FC and EC measures extracted through the MOU-EC model. For the between-group classification, we used a further set of SC features. First, we transformed the FC, SC, and EC matrices estimated by the model for each resting-state session into a vector by selecting the lower triangle of the FC and SC matrices and by applying the SC mask to the EC matrix. The resulting features arrays consisted of a vector of 3874 EC links and other 2 vectors of 6670 for FC and SC links. Then, we normalized the feature arrays by z-scoring within each session the FC, SC, and EC connections. We used the normalized FC and EC (and SC for the between-group classification) vectors of each sample (i.e. subject) to train 2 different classification algorithms, namely the 1-Nearest Neighbor (1NN) and the linear SVM. We applied the 1NN using the Pearson correlation coefficient as a metric to predict the class of the sessions belonging to the test-set by identifying the most similar session from the train-set. Therefore, we used the 1NN to capture the overall similarity between resting-state sessions of EC, FC, and SC profiles. On the other hand, we applied the SVM classifier to allow for feature selection procedures, highlighting the specific EC links that most contributed to the correct classification of resting-state sessions. Indeed, the SVM uses as a metric the sample’s distance from the separating hyperplane that represents a decision boundary by partitioning the feature space into one set for each class. To fit the hyperplane that optimally divides the 2 classes (i.e. pre- vs post-training rs sessions or NFB vs CLT subjects), the SVM determines a linear predictor function that combines weights for each input feature.

We performed cross-validation of the 1NN and the SVM by applying a random splitting procedure in which the 80% of samples were used to train the classification algorithms and the remaining 20% for testing his performance. We repeated the random splitting procedure 50 times in order to assess the impact of different splits of data in train/test sets on 1NN and SVM performances. To evaluate the models performances in distinguishing between resting-state sessions, we only considered the accuracy of the 50 predictions on unknown data belonging the test-set.

To assess whether some metrics (FC, SC, or EC) or classification algorithms (1NN or SVM) performed better, we statistically compared accuracy distributions using the Wilcoxon rank-sum method. Moreover, we tested whether the models performances were significantly above changes by statistically comparing accuracy distribution of real-labeled data with the performance of surrogate data (i.e. randomly shuffled labeled data).

#### Signatures extraction through SVM-RFE

Finally, we aimed to investigate which were the NFB-induced changes in specific EC links that differentiated the pre- and post-training resting-state sessions within the NFB group. Therefore, we performed Recursive-Feature Elimination using the SVM (SVM-RFE) to rank the features according to their importance for classification performance (Guyon et al. 2002). This procedure allows for feature ranking by selecting at each step a different subset of features and pruning the least relevant ones, until only the most important links are left and used to assess classification performance. We repeated the SVM-RFE procedure 10 times by randomly selecting at each iteration the 80% of samples to train and the 20% for testing the model performance using a subset of features for which mean accuracy was maximum, and adding further features provided no improvement to the performance.

### Intrinsic-Ignition Framework

We applied the Intrinsic-Ignition framework to characterize differences between the 2 groups and between the resting-state sessions of each group (pre- vs post-training) in the spatiotemporal transmission of information across the brain. First, we analyzed spatiotemporal dynamics within the subnetwork highlighted by the SVM-RFE comprising 42 brain regions. Then, to understand training-related effects on the entire brain network, we repeated the analysis at the whole-brain level. This data-driven approach captures the effect of spontaneous local activity on the global computation, reflecting the level of integration with the rest of the brain network (Deco and Kringelbach 2017). Therefore, this analysis informs about the hierarchical information processing profile that characterizes a given brain state. The algorithm to calculate the intrinsic integration for all the events elicited by a brain area within a given time-window is shown in Fig. 1C. In brief, we first computed the Hilbert Transform of the filtered time-series (band-pass filter of 0.04–0.07 Hz). Then, the phase lock matrix $P_{jk}(t)$ was calculated, which describes for each time-point the state of pair-wise phase synchronization $t$ between regions $j$ and $k$. The mathematical equation to calculate the phase lock matrix is as follows:

(1) \begin{align*}& P_{jk}(t)=\mathrm{e}^{-3 | \varphi_{j}(t)-\varphi_{k}(t)|} \end{align*}

Here, $\varphi _{j}(t)$ and $\varphi _{k}(t)$ represent the obtained phase of the brain regions $j$ and $k$ at time $t$. To capture events within a given brain area, a threshold $\theta $ (given by the sum of the mean and the standard deviation of the signal in each brain region) was fixed and the timeseries was transformed into z-scores, $z_i$(t) (Tagliazucchi et al. 2012). Therefore, we binarized the symmetric phase lock matrix $P_{jk}(t)$ by defining a value of 0 if $|P_{jk}| <$$\theta $ and 1 otherwise. Lastly, we averaged all the synchronized events within each fixed time-window of 4-TR and we computed the integration value as the largest subcomponent of the binarized phase lock matrix. The integration value reflects the broadness of communication across the entire brain network. Finally, after repeating this process for each driving event, we computed the Intrinsic-Driven Mean Integration (IDMI) and its variability (i.e. metastability), given by the mean and the the standard deviation of the integration value across all events, respectively.

## Results

The effects of NFB training on motor brain areas, functional and structural connecitivity, and motor behavior have been published previously (Marins et al. 2015). In brief, we found that less than 1 h of NFB training allowed the control of brain signals and reinforce motor execution-related brain patterns in the absence of overt movement (while performing MI alone), which resulted in a strengthening of both sensorimotor network (SMN) and DMN FC and increase of mean FA of sensorimotor segment of corpus callosum. Nonetheless, the impact on motor performance did not seem to depend on NFB training.

### Classification of resting-state sessions within each group

**Fig. 2:**
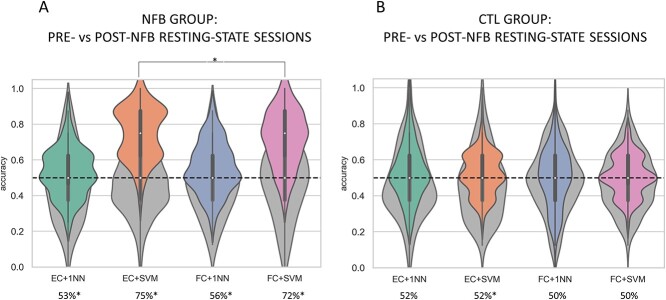
**Classification results of pre- vs post-training resting-state sessions within each group.** Classification performance according to features and classification algorithm is shown for the NFB group (A) and controls (B). The green violin plots represent accuracies distributions for the 1NN classifier combined with EC features, while the blue ones show 1NN performance using FC features. Accuracies related to SVM are shown in orange for EC and in pink for FC features. The gray violin plots in the background show accuracy distributions for each metric and classifier using surrogate data. Only for the NFB all the combinations between classifiers (SVM and 1NN) and type of features were significantly higher than chance-level (*P*<.05). Furthermore, the best accuracy in the NFB group was yielded by the SVM using EC features (mean accuracy = 75%), which was significantly higher than performance reached using FC features. In contrast, for the control group performance was not significantly higher than chance-level (*P*>.05), except for the combination EC+SVM which reached significance (*P*=.01) but showed a moderate average accuracy of 52%.

We investigated whether the post-training (real or sham) resting-state session could be accurately differentiated from the pre-training resting-state session. We applied 2 machine-learning algorithms, the 1NN and the SVM, to classify resting-state sessions, separately for each group (i.e. NFB and CTL group). We performed statistical comparisons of accuracy distributions in order to highlight which type of features and classifier performed better. Moreover, to understand whether the performances of the 2 ML models were above chance-level, we compared real accuracy distributions with performances using surrogate data. Results of classification for each group are presented in Fig. 2.

For the NFB group, classification using EC features with SVM yielded an average accuracy of 75%, significantly higher than the accuracy reached when using FC features (average accuracy = 72%, *P* =.02). This result suggests that it is possible to differentiate resting-state brain dynamics between pre- and post-training for subjects who underwent NFB training, and that EC features captured the characteristic connectivity changes induced by NFB training better than FC. In contrast, classification using 1NN led to a lower performance, with no significant difference (*P*>.05) between EC (average accuracy =53%) and FC (average accuracy =56%) features, indicating that both the global EC profile and FC patterns were overall similar between the 2 resting-state sessions in the NFB group. Nonetheless, performance of all combination of metrics and classifiers were significantly above chance (*P*<.0001 for EC+SVM, FC+1NN, FC+SVM, and *P*=.008 for EC+1NN) in the NFB group.

On the other hand, classification performance for the CTL group was around chance-level for all metrics and classifiers and did not reach significance (*P*>.05) when compared with accuracy distributions using surrogate data, except for the combination EC+SVM (average accuracy= 52%, *P*=.01). These results suggest that the sham training did not induce changes in the global EC and FC profiles, nor localized changes in FC patterns.

### Signature extraction of NFB training: support network

Since SVM performance using EC features led to a high accuracy in the NFB group, we subsequently performed SVM-RFE to investigate which were the localized EC links (i.e. support network) that contributed the most to the accurate distinction between pre- and post-training resting-state sessions.

**Fig. 3:**
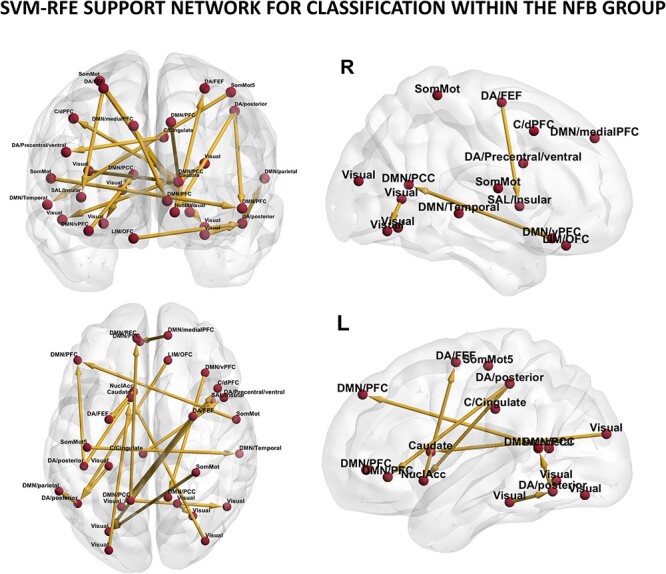
**Results of SVM-RFE in NFB group: support network for the classification of pre- and post-training resting-state sessions.** The signatures of NFB training (i.e. highest-ranked EC links) are shown in axial, coronal, and sagittal views both for the right and left hemispheres. The 7 functional networks are drawn from the parcellation in 100 nodes (Schaefer et al. 2018) together with 16 subcortical regions (Tian et al. 2020). Only nodes belonging to the support network are shown in the rendered brain. Node labels are provided including the name of the functional network they belong to. The yellow arrows show the directionality of connection between regions (i.e. EC edges). Abbreviations for networks: DA (Dorsal Attention), SomMot (Somatomotor), SAL( Salience/ventral attention), DMN (Default Mode), C (Control), LIM (Limbic). Abbreviation for brain areas: PFC (Prefrontal cortex), dPFC (Dorsal prefrontal cortex), vPFC (ventral prefrontal cortex), OFC (Orbitofrontal cortex), PCC (Posterior cingulate cortex), FEF (Frontal eye field), NuclAcc (Nucleus accumbens). Visualization of results was generated using the BrainNet Viewer Toolbox (Xia et al. 2013).

The NFB-induced signatures extracted through SVM-RFE are shown in Fig. 3. We found that the support network comprised 21 edges distributed across the whole brain and mostly involved connections within and between the DMN, dorsal attention, visual, somatomotor, and control networks. Moreover, the feature selection procedure highlighted the importance in distinguishing connectivity patterns between the pre- and post-training resting-state sessions of connections from areas involved in attentional and executive functions toward subcortical areas (i.e. globus pallidus and nucleus accumbens) and regions belonging to the DMN. In particular, the posterior regions belonging to the dorsal attention network appeared to be a central hub, exerting top-down regulation over both frontal and parietal areas belonging to the DMN, to the frontal operculum/insular region (part of the salience/ventral attention network), to the nucleus accumbens and to the left visual areas. At the same time, we found that the DMN also received top-down regulation from the somatomotor network and that it was the only large-scale network showing within-network connections between its posterior and frontal areas. Lastly, the 2 subcortical areas, the globus pallidus and nucleus accumbens, respectively, involved in the regulation of voluntary movements and in the processing of reward, showed both top-down connections from visual and dorsal attention areas and projections to the prefrontal portion of the dorsal attention network. Altogether, these findings suggest that the most relevant features that allowed discriminating between pre- and post-training sessions in the group that underwent NFB training involved connections between several large-scale networks, going beyond the solely selected area and network (i.e. somatomotor) that was trained during the NFB session.

### Group classification during post-training resting-state session

To investigate whether connectivity patterns could be precisely distinguished between groups, we classified the post-training resting-state session of both groups using the same procedure as explained in the method section. Again, we statistically tested the significance of accuracy distributions against the model performance using surrogate data.

Since a previous study published with this dataset (Marins et al. 2019) found a significant difference in SC between the 2 groups in the post-training session, we included a new set of features consisting of the vectorized SC matrix specific to each subject obtained from the post-training DTI images. Therefore, we compared model performance using EC, FC, and SC features.

**Fig. 4:**
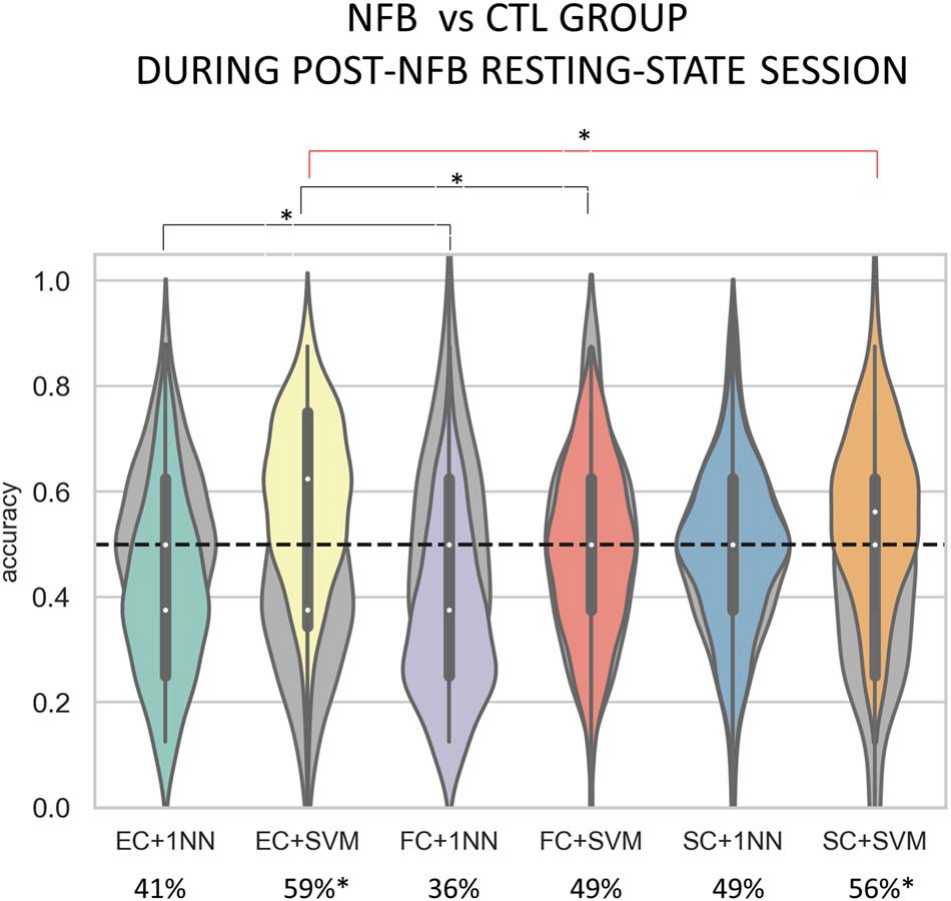
**Accuracy distributions of group classification of resting-state sessions acquired post-training.** Performances of 1NN and SVM are shown for each of the 3 feature sets. The green and yellow violin plots show accuracy distribution when using EC features for 1NN and SVM, respectively. Performance using FC features is colored in violet for 1NN and in red for SVM, while the blue (1NN) and orange (SVM) violin plots depict accuracy distribution using SC features. Lastly, the gray plots in the background reflect performance of each metric and classifier when using surrogate data. We found that using EC features with SVM classifier led to the best performance in distinguishing the 2 groups, yielding significantly higher average accuracy as compared with FC features and sightly improving performance as compared with SC features. Performances of 1NN was significantly different depending on the type of features, with EC yielding the best average accuracy. However, only performances of SVM were significantly above chance-level, except for FC features (*P*>.05).

Results of group classification during post-training resting-state are shown in Fig. 4.

We found that the best performance was reached using EC features and the SVM (average accuracy=59%), outperforming FC (average accuracy=49%; *P*=.02), and sightly improving performance compared with SC features (average accuracy=56%) but the difference did not reach significance (*P*>.05). This result suggests that EC weights, which hold anatomo-functional information, are more informative than FC alone when distinguishing between subjects belonging to 2 different groups. Performance of 1NN also significantly improved when using EC features (average accuracy=41%) compared with FC (average accuracy=36%; *P*<.0001) but there were no significant differences with performance using SC features (*P*>.05). However, performance of 1NN was not significantly higher than chance-level for any of the 3 feature sets (*P*>.05). Since the 1NN uses the Pearson correlation as a similarity measure, poor 1NN performance suggests that the global EC, FC, and SC profile was overall similar in the 2 groups. By contrast, SVM performance was significantly above chance-level for for EC (*P*<.0001) and SC features (*P*<.0001), but not for FC features (*P*>.05). Together these results indicate that there are structural differences between the 2 groups, thus making EC better suited for discriminating subjects belonging to the NFB or the CTL group, and that the distinction between the 2 groups relies on localized weights of some specific EC links instead of the global EC profile.

### Intrinsic Ignition profiles in the support network and at the whole-brain level

Following the results of classification and SVM-RFE, we performed statistical analysis of the Intrinsic-Driven Mean Integration (IDMI) and the variability of intrinsic-driven integration comparing the 2 resting-state sessions (i.e. pre- and post-training) of both groups in order to assess effects of NFB on brain’s functional dynamics. First, we investigated whether these 2 measures were significantly different within the support network highlighted by the feature selection procedure. Then, we repeated the analysis including all brain regions in order to understand the impact of NFB training on functional dynamics at the whole-brain level.

**Fig. 5:**
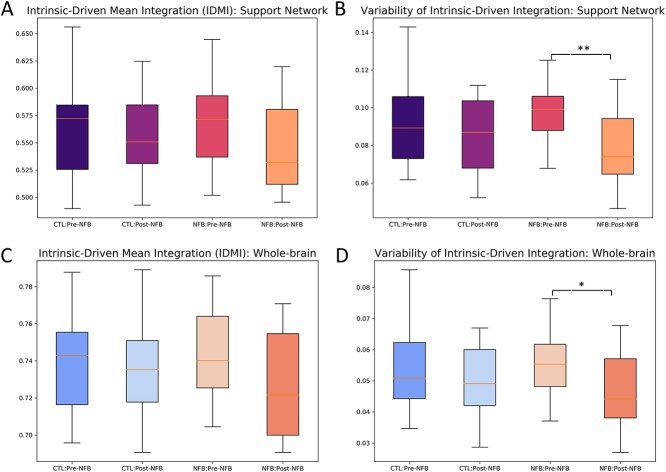
**Intrinsic ignition profiles for both groups in the support network and at the whole-brain level.** The boxplots show the mean IDMI (A) and variability of IDMI (B) values within the support network for each resting-state session of both groups. Boxplots in Figs. (A and B) colored in dark blue represent values of the resting-state session before the sham training, while the purple ones represent values of the post-training sessions for the CTL group. Mean IDMI and its variability are shown in red and in orange for the NFB group, for the pre-NFB and post-NFB resting-state sessions, respectively. We found significant differences in the variability of the IDMI for the NFB within the support network (*P*=.01), with the post-NFB sessions showing decreased metastability compared with the pre-NFB session. Figures C and D show IDMI and metastability values at the whole-brain level. Boxplots in blue and lighter blue represent values of the CTL group for the pre- and post- (sham) training, respectively. The light and dark orange boxplots show values of the pre- and post-NFB training in the NFB group. Again, we found the same effect of reduced variability (i.e. metastability) at for the post-NFB session in the NFB at the whole-brain level (*P*=.03). There were no significant differences within the CTL group for any of the 2 measures nor within the support network or at the whole brain.

Figure 5A and B show IDMI and the variability of intrinsic-driven integration for both groups and resting-state sessions with respect to the support network. Figure 5C and 5D represent intrinsic ignition profiles at the whole-brain level for both groups and resting-state sessions.

We report no significant differences in IDMI measures for all comparisons both in the support network (Fig. 5A) and at the whole-brain level (Fig. 5C), indicating that the spatial diversity of functional dynamics were similar across groups and resting-state sessions. By contrast, we found that in the regions highlighted by the RFE support network the variability of intrinsic-driven integration, which represents the heterogeneity of dynamics of a given brain area (i.e. metastability), was significantly decreased in the post-training resting-state session in the NFB group (*P*=.01) but not in the CTL group (*P*>.05). The variability of intrinsic-driven integration was also significantly lower at the whole-brain level (Fig. 5D; *P*=.03) during the post-training resting-state compared with the pre-NFB session in the NFB group. Again, no significant differences were observed between groups nor between resting-state session within the CTL group. These results suggest that NFB training induced decrease dynamical complexity (i.e. switching between different brain states) as captured by the variability of intrinsic-driven integration measure, and that this effect was more pronounced when analyzing only the 42 regions of the support network compared with the whole brain.

## Discussion

In this work, we aimed to provide a causal mechanisms underlying NFB training by exploring whole-brain differences in resting-state EC between participants who underwent motor-imagery NFB training and control subjects belonging to the sham condition. We used a computational model and a data-driven method to analyze functional and structural data of a randomized, double-blind, and sham-controlled study (Marins et al. 2015) in order to extract EC estimates and empirical measures of functional dynamics. By applying machine-learning tools, we provided evidence that a single session of NFB training led to changes in brain’s EC that went beyond the trained (sensorimotor) areas. More importantly, we demonstrated that only participants who received real feedback based on their own brain activity showed differences in information propagation and functional dynamics. In contrast, the CTL group did not show any significant training-related changes in EC or in whole-brain functional dynamics.

We applied a generative model, the MOU-EC, to reproduce lagged and non-lagged second order statistics of resting-state data and to infer EC by combining both structural and functional information. First, we performed within-group classification between the pre- and post-training resting-state sessions using EC and FC features to investigate functional changes induced by the NFB or sham training. Then, we compared the post-training resting-state sessions of the 2 groups (NFB and CTL groups) to assess for differences in structural, functional, and effective connections depending on whether participants underwent real or sham training. We found that for the NFB group it was possible to discriminate with high accuracy the pre- and post- resting-state sessions, and that EC features were more informative than the FC ones. In particular, we showed that EC led to a higher performance than FC when using the SVM, while differences in performance depending on the type of features were not significant for the 1NN classifier. This result suggests that the NFB training induced localized changes in connectivity patterns that were better captured by EC estimates as compared with FC. In contrast, classification performance for the CTL group was not above chance-level (best accuracy = 52% using SVM+EC), indicating that the sham training did not lead to significant changes in brain’s information propagation. Results of between-group classification also emphasizes the predictive power of EC features, leading to higher performance than FC features in distinguishing subjects belonging to 2 different groups. Taken together, these results confirmed our hypothesis that EC, which holds information about the underlying anatomical connectivity together with the functional dynamics, is a better predictor for discriminating characteristic connectivity patterns related to NFB training.

To fulfill the purpose of providing a causal mechanisms underlying NFB training, we applied a feature selection procedure to highlight the direct connections between brain regions that were the most relevant for the classification of pre- and post-training sessions within the NFB group. We found that the signatures of NFB training comprised brain areas that were not directly trained through the NFB protocol, indicating that NFB induced functional neuroplasticity that went beyond the targeted area. Specifically, results of SVM-RFE highlighted a subnetwork comprising 21 distributed edges that showed training-induced changes and involved nodes belonging to the default-mode, dorsal attention, visual, somatomotor and control networks, corroborating and extending the findings obtained in the previous publication with the present dataset (Marins et al. 2019). Together with these large-scale networks, the globus pallidus and the nucleus accumbens were involved in this support network. These 2 subcortical regions received top-down regulation from areas belonging to the visual and the dorsal attention network while at the same time sending projections to the prefrontal areas, part of the dorsal attention network. We showed that the posterior areas of the dorsal attention network were at the core of this subnetwork, sending top-down information to the prefrontal and parietal regions of the DMN, to the insular region, to the nucleus accumbens and the left visual areas. While most of the edges involved between-network direct links, we found that the DMN was the only large-scale network showing within-network connections, with parietal areas sending information to the frontal ones. These results are in line with previous literature exploring NFB-induced changes in resting-state networks. In particular, the involvement of both the dorsal (i.e. the globus pallidus) and the ventral (i.e. the nucleus accumbens) striatum in the learning process underlying NFB training has been previously widely documented (Johnston et al. 2010; Koralek et al. 2012, 2013; Ramot et al. 2016, for a review see Sitaram et al. 2017). Furthermore, in a meta-analysis, Emmert and colleagues proposed that the striatum together with the insula, also part of the support network highlighted by SVM-RFE, constitute a “regulating network” by being, respectively, involved in reward-based learning and self-awareness (Emmert et al. 2017). Evidence about the involvement of areas belonging to the dorsal attention and executive networks in NFB training have also been reported (Emmert et al. 2016; Gevensleben et al. 2014; Ninaus et al. 2013), as well of visual areas when a visual feedback is presented (Murray and Wojciulik 2004). Furthermore, effects of NFB training on the DMN connectivity have been proved by several studies (Kluetsch et al. 2014; Nicholson et al. 2018; Rubia et al. 2019; Zweerings et al. 2019). Here, we went beyond FC analysis by showing changes in direct connections (akin to EC) between these large-scale networks and the striatum, providing a causal mechanisms underlying NFB training.

Finally, to assess whether changes in information propagation were also reflected in the brain’s spatiotemporal dynamics, we applied a data-driven approach to analyze the resting-state fMRI data. We demonstrated that for the NFB group, the post-training resting-state session was characterized by a reduced dynamical complexity (i.e. less switching between different brain states), both at the whole-brain level and within the support-network highlighted through the feature selection procedure. Again, we did not find any significant difference in brain dynamics for the CTL group. These results suggest that only NFB training leads to a more metastable states in the post-training resting-state session, restricting the dynamical repertoire to a less frequent switching between different brain states. These findings are in line with effects of meditation training on the brain’s dynamical complexity. In their work, Escrichs et al. (2019) have investigated differences in functional dynamics between meditators and controls using the same data-driven approach used here. They found that both groups, with a greater effects in the meditator group, showed reduced variability in the dynamic repertoire during meditation condition as compared with resting-state.

Altogether, this study demonstrated that a single session of NFB training on motor imagery can influence brain’s information propagation and its spatiotemporal dynamics. Furthermore, here we provided evidence of the causal mechanisms selectively related to NFB training with real feedback, highlighting the importance of localized changes in information processing, which were not observed when participants received sham feedback. An interesting perspective would be to explore different NFB protocols targeting brain areas unrelated to the SMN to assess whether the connectivity changes found here are shared among different training protocols. It is worth noting that we assessed the resting-state sessions acquired immediately after the NFB training. Future studies should investigate whether these effects remain more than only a few minutes after training. Also, given its capability to predict behavioral outcomes (Adhikari et al. 2020), EC measurements should also be explored as possible predictors of NFB performance, thus helping to tailor more effective designs.

### Conclusions

In this secondary analysis of a previous study (Marins et al. 2019), we corroborated and extended the previous findings by elucidating a causal mechanism underlying NFB training that goes beyond functional correlation between brain regions. In particular, by estimating whole-brain EC profiles through a dynamical model, we showed that one session of NFB training can lead to functional and structural neuroplasticity during resting state. We demonstrated that NFB training led to localized changes in brain’s functional dynamics, and that these effect was better captured by EC measures as compared with standard FC. Furthermore, we found that NFB training-related signatures identified through RFE, included directed connectivity between brain areas involved in reward processing and implicit learning, together with regions belonging to the somatomotor, control, default mode, and attention networks. Lastly, we showed that NFB training led to a decreased dynamical complexity (i.e. metastability) at both whole-brain and support-network levels, which was not observed in the CTL group that received sham feedback. Taken together, our findings provide new evidence of whole-brain plasticity elicited by NFB training at the structure-function level. We proposed a causal mechanism underlying NFB that may have foremost implications for potential improvement of training protocols, and ultimately allowing to evaluate the clinical relevance of NFB for neuropsychiatric conditions in which brain connectivity is altered.
